# Genetics and Phenotypes of Late-Onset Neurodegeneration in Neurodevelopmental Disorders

**DOI:** 10.7759/cureus.93530

**Published:** 2025-09-30

**Authors:** Jamir Pitton Rissardo, Fatemeh Rashidi, Masoumeh Rashidi, Meryem Bahar, Kimia Kheirandishdoolabi, Ana Leticia Fornari Caprara, Farbood Khademhamzeh, Sogand Ranjbar, Omesh Prathiraja, Maleesha Jayasinghe

**Affiliations:** 1 Neurology, Cooper University Hospital, Camden, USA; 2 Medicine, Nanjing Medical University, Nanjing, CHN; 3 Medical Education, Nanjing Medical University, Nanjing, CHN; 4 Neurology, Universidade Federal de Santa Maria, Santa Maria, BRA; 5 Orthopedic Surgery, Nanjing Medical University, Nanjing, CHN; 6 Medical Imaging, Nanjing Medical University, Nanjing, CHN; 7 Medicine and Surgery, Nanjing Medical University, Nanjing, CHN

**Keywords:** early developmental phenotype, genetic predisposition, mitochondrial dysfunction, neurodegeneration, neurodevelopmental disorders

## Abstract

Late-onset neurodegenerative diseases may originate from subtle vulnerabilities established during early development, yet the specific mechanisms linking early-life factors to later pathology remain poorly understood. Emerging evidence suggests that prenatal and postnatal influences, including genetic predispositions, maternal health, and environmental exposures, shape neural circuits in ways that may predispose individuals to neurodegeneration. Subclinical abnormalities in synaptic pruning, neurogenesis, and immune regulation are increasingly recognized as latent risk factors, but their precise contribution to disease onset has not been clearly defined. Advances in biomarker discovery, particularly in proteomic, genetic, and imaging domains, offer promising opportunities to detect such vulnerabilities before clinical symptoms arise. Despite these insights, the field lacks longitudinal and mechanistic studies that connect early developmental disruptions to specific neurodegenerative outcomes. Addressing this gap will require integrative research that combines experimental models with life-course studies to clarify causal pathways. By identifying concrete mechanistic links, future work may inform more targeted preventive strategies, rather than broadly proposing reductions in global disease burden.

## Introduction and background

**Figure 1:**
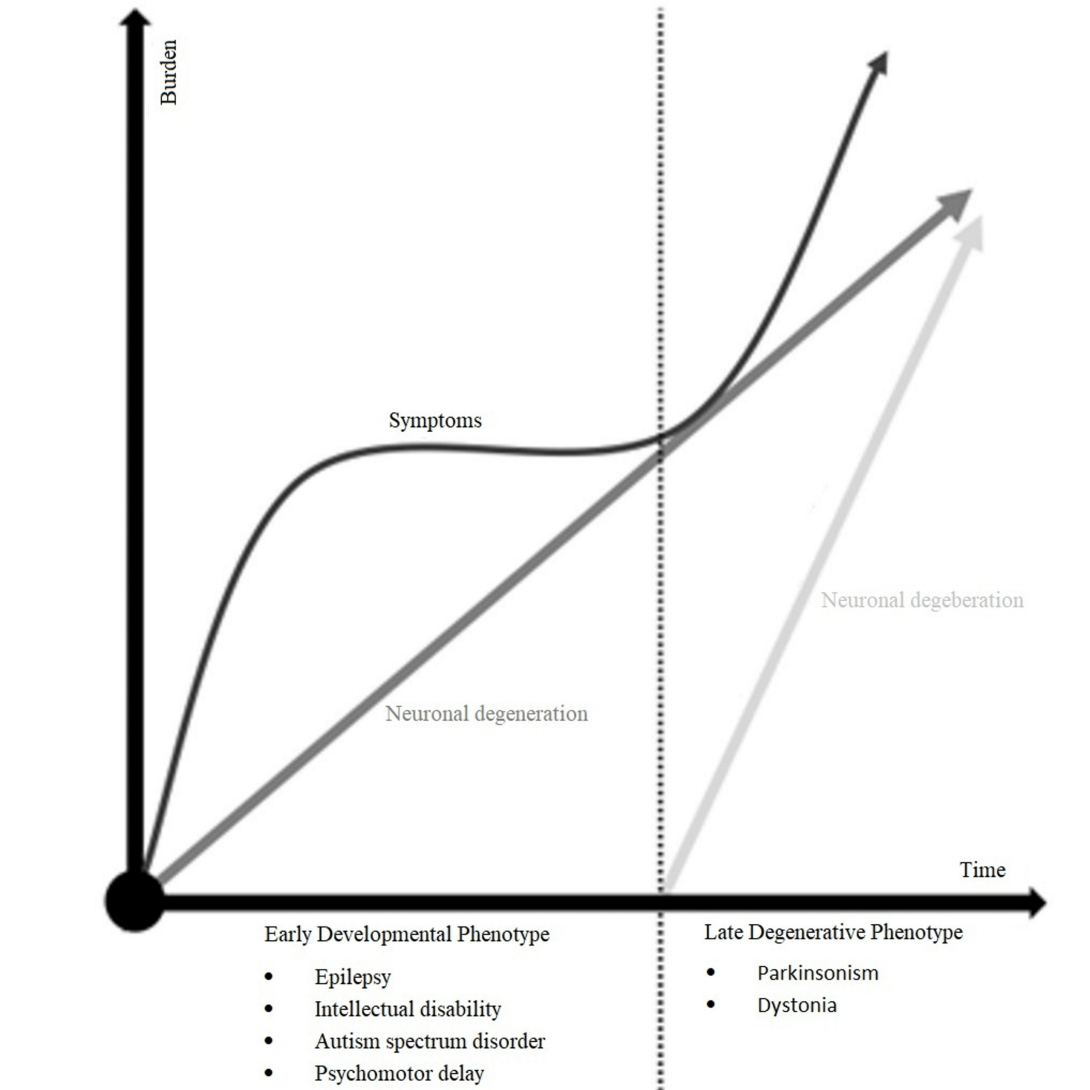
Late-onset neurodegeneration in neurodevelopmental disorders Original figure created by the authors.

Neurodevelopmental disorders (NDDs) are a heterogeneous group of conditions characterized by atypical brain development and function, often manifesting as cognitive, behavioral, or motor impairments. While traditionally viewed as static conditions, recent evidence has challenged this paradigm, highlighting the potential for late-onset neurodegenerative processes in certain NDDs [1]. This emerging area of research bridges the fields of neurodevelopment and neurodegeneration, revealing complex biological underpinnings that may contribute to progressive neurological decline in individuals with NDDs (Figure 1).

The intersection of neurodevelopment and neurodegeneration raises critical questions about the temporal dynamics of brain embryology. Ruth M. Meredith highlighted that sensitive and critical periods shape neural circuit formation and that disruptions during these windows can leave lasting vulnerabilities that predispose individuals to later neurological dysfunction [2]. Meanwhile, W. Gordon Johnson proposed that late-onset neurodegenerative diseases often arise from the gradual accumulation of insoluble proteins, which may interact with or unmask these early-established vulnerabilities [3]. Consistent with these perspectives, longitudinal studies have increasingly documented that some individuals with NDDs exhibit new or worsening neurological symptoms later in life, including motor dysfunction, cognitive decline, and psychiatric disturbances. This suggests that the trajectory of NDDs may extend beyond childhood, encompassing phases of neurodegeneration that unfold during adolescence or adulthood.

The molecular and cellular mechanisms driving the transition from NDDs to late-onset neurodegeneration remain poorly understood but are likely multifactorial. While dysregulated synaptic plasticity, mitochondrial dysfunction, oxidative stress, and chronic neuroinflammation are established contributors [4], their specific roles in late-onset trajectories require deeper investigation. For instance, chronic neuroinflammation, characterized by persistent microglial activation and elevated pro-inflammatory cytokines, may progressively damage vulnerable neuronal circuits established during development, ultimately leading to functional decline [5]. Genetically, common variants in microglial-enriched genes, such as *TREM2* [6] and those in the complement cascade (e.g., *C4A*) [7], which are well-known risk factors for neurodegenerative diseases, appear to be particularly significant in modifying the late-onset risk in individuals with NDDs. Environmentally, metabolic stress from mid-life obesity and type II diabetes [8], as well as exposure to airborne environmental toxicants like fine particulate matter (PM2.5) [9], are emerging as potent exacerbating factors that can interact with genetic vulnerabilities to disrupt protein homeostasis and accelerate neuronal loss, thereby tipping the balance toward neurodegeneration in susceptible individuals [10].

This emerging framework has profound implications for clinical management and therapeutic development. Recognizing the potential for late-onset neurodegeneration in NDDs necessitates a paradigm shift in how these disorders are monitored and treated over the lifespan. Current clinical practices often focus on addressing early developmental deficits, with less emphasis on long-term neurological outcomes. Integrating neuroprotective strategies, such as targeted pharmacological interventions and lifestyle modifications, into the care of individuals with NDDs may help mitigate the risk of neurodegeneration and improve quality of life.

## Review

Genetic insights into neurodegeneration

Common Neurodegenerative Disorders (Alzheimer’s Disease, Huntington's Disease, and Parkinson’s Disease)

Genetic mutations that occur during embryogenesis can lead to neurodevelopmental abnormalities, including stem cell loss, altered cell numbers, and inadequate identity programming (e.g., altered neurotransmitter and receptor profiles). These initial defects can subsequently result in migration defects, reduced axonal growth, dendritic growth alterations, altered synaptogenesis, suboptimal plasticity and learning, and diminished neuronal network integrity [11]. In this way, the development of late-onset neurodegeneration depends on "priming" effects in patients with impaired compensatory systems in the brain and with increased susceptibility to other hits during life, including genetic and non-genetic risk factors. Interestingly, these effects can be observed in induced pluripotent stem cell (iPSC)-derived and adult stem cell (ASC)-derived (also known as patient-derived organoids, PDOs) models [11]; for instance, cerebral organoids carrying LRRK2 G2019S mutations exhibit early developmental defects such as abnormal autophagy, lysosomal dysfunction, and synuclein pathology, while those overexpressing wild-type alpha-synuclein show impaired neurogenesis and neuronal maturation, providing a direct link between neurodevelopmental deficits and later neurodegenerative phenotypes [12-14].

A possible mechanism to explain neurodegeneration is a diphasic course, in which compensatory mechanisms initially preserve neural function but become exhausted over time, ultimately giving rise to a degenerative phase. Evidence for such trajectories has been suggested by longitudinal observations in human cohorts, as well as experimental findings in animal models, although the concept remains partly theoretical and requires further validation [15].

Another possible explanation is age-related changes leading to abnormalities in brain resilience pathways. Moreover, a space-temporal shift in the expression of the causative gene, with experimental evidence for *FOXG1* and *FRRS1L*, has been observed, showing different expressions at various stages of life [16]. Lastly, chronic brain damage induced by symptoms like seizures in dramatic encephalopathy can also lead to neurodegeneration.

Rare Neurodegenerative Disorders With Later-Onset Neurodegeneration

There is a diphasic course of later-onset neurodegeneration in an early developmental phenotype.

BPAN and WDR45

The initial case description of later-onset neurodegeneration was with BPAN (beta-propeller protein-associated neurodegeneration) in a case of *WDR45* (WD repeat domain phosphoinositide-interacting protein 4, WIPI4), leading to neurodegeneration with brain iron accumulation (NBIA), which is a distinct disorder not only phenotypically but also genetically. It is an X-linked disorder that mainly manifests in females since they have two copies of the X chromosome, in which one of the X chromosomes compensates for the deficiency; on the other hand, males affected by this disorder have severe features rarely achieving adulthood, and those that achieve adulthood have mosaicism [17]. Interestingly, the historical name of this disorder was SENDA (static encephalopathy of childhood with neurodegeneration in adulthood), which is the phenotypic description. At the imaging level, this disease is distinctive among NBIA forms due to the pronounced hypointensity in the substantia nigra relative to the striatum, along with a surrounding halo of hypointensity.

*WDR45* codes for a protein with the same name, "WDR45," in which WD refers to tryptophan (W) and aspartic acid (D). It is part of the WIPI proteins with phosphatidyl-inositol interaction with membranes in the cell, and there are four members in this family: WIPI1, WIPI2, WIPI3, and WIPI4. The WD domain has the shape of a propeller of 7WD and 40 domains, making molecular scaffolds (fingers) that place different protein complexes together. The WDR45 is essential to scaffold two other proteins related to autophagy, ATG9 and ATG2, which transport lipids to various compartments in the cells. Therefore, the new phospholipids go through ATG2 from the endoplasmic reticulum to the nascent phagophore organelle. Thus, WIPI family defects lead to defects in autophagy. However, WIPI4 has the lowest severity, so there are doubts that WIPI4's most crucial function is related to autophagy compared to the other parallel proteins [18]. These processes occur in the cytosol and synaptic area, where, in the synaptic cleft, they maintain protein quality due to the extended distance from the cell body nucleus [19].

WIPI4 may have a different function in neurodegeneration besides autophagy, which is related to ferroptosis [20]. This is a hot topic in common neurodegenerative disorders, including Alzheimer's disease and Parkinson's disease, and for the opposite reason for chemotherapeutic drug targets. Phosphatidylserine is transported to the mitochondria from the endoplasmic reticulum and other membranes, such as the mitochondria-associated membrane, with the WIPI4-ATG2 mechanism. The mutation of WIPI4 may lead ATG2 to link a direct pathway between the endoplasmic reticulum and mitochondria without stabilization, leading to lipid peroxidation (from phosphatidylserine to phosphatidylethanolamine) and, eventually, cell death. Therefore, the WIPI4 function is a buffer for ferroptosis.

PP2A Complex, PPP2R5D, and PTPA

Protein phosphatase 2A (PP2A) is a critical phosphatase in the brain, essential for dephosphorylating multiple proteins involved in fundamental neural processes such as learning, memory, synaptic plasticity, and protein quality control. It also regulates neuronal stress responses, cell cycle progression, and enzymes like tyrosine hydroxylase, while controlling the phosphorylation state of neurodegeneration-associated proteins such as alpha-synuclein, LRRK2, and MAPT [21]. PP2A functions as a heterotrimeric complex composed of scaffold (A), regulatory (B; including B, B’, B’’, B’’’), and catalytic (C) subunits. The B’ subunit family includes PPP2R5A-PPP2R5E, and variants in PPP2R5D have been reported in early-onset parkinsonism (Table 1) [22-27].

**Table 1:** Early-onset Parkinson's disease in subjects with PPP2R5D mutation ^a^: Number of individuals reported ^b^: Age of onset of parkinsonism symptoms, expressed in years EOPD: early-onset Parkinson's disease, NA: not available Original table created by the authors.

Reference	N^a^	Age^b^	Mutation	Note
Kim et al. (2020) [22]	3	25-40	p.E200K heterozygous	Probably a de-novo mutation in one patient. Mild developmental delay and levodopa-responsive parkinsonism.
Hetzelt et al. (2021) [23]	1	25	p.E198K de-novo	Intellectual disability, hypotonia, and seizures; progressive motor decline and levodopa-responsive parkinsonism.
Walker et al. (2021) [24]	1	20	p.E250K de-novo	Delayed early developmental milestones, macrocephaly, moderate intellectual disability, attention-deficit/hyperactivity disorder, and auditory processing disorder.
Ning et al. (2022) [25]	2	NA	p.R91S,p.R523L	2/145 EOPD patients, missense variants.
Mahale et al. (2024) [26]	1	32	p.E200K	Levodopa-responsive parkinsonism; No neurodevelopmental delay, intellectual disability, or autism spectrum disorder.
Yau et al. (2024) [27]	1	35	p.E200K de-novo	Mild developmental motor delay and intellectual disability; levodopa-responsive parkinsonism.

Another key regulator of this complex is phosphotyrosyl phosphatase activator (PTPA), which activates PP2A by counteracting its inhibitor pectin methylesterase 1 (PME1, also known as pectinesterase 1). PTPA deficiency leads to the accumulation of inactive PP2A and has been described in two families showing intellectual disability and very early-onset neurodevelopmental delay, followed two decades later by parkinsonism responsive to levodopa [28]. Similarly, a report from China described a patient carrying a homozygous p.Met329Val variant who developed early-onset Parkinson's disease without a preceding neurodevelopmental phase [29]. These findings suggest that PP2A dysfunction can first disrupt neurodevelopment and later predispose neurons to degeneration, positioning it as a mechanistic bridge between Neurodevelopmental disorders and Neurodegenerative diseases.

Synaptopathies (DNAJC6, SYNJ1, RAB39B)

Many genes and monogenic disorders affect synapses (changed from synapsis), and three will be discussed. DnaJ heat shock protein family (Hsp40) member C6 (DNAJC6) encodes auxilin, a neuronal heat shock protein 70 (HSP70) co-chaperone. *DNAJC6* mutations are known to cause autosomal recessive juvenile atypical parkinsonism (PARK19). The initial description was in a Palestinian family with juvenile parkinsonism (onset < 20 years) and levodopa-unresponsive symptoms identified by whole-exome sequencing [30]. This report was followed by a Turkish family with juvenile parkinsonism in which mental retardation, pyramidal signs, seizures, and partial levodopa responsiveness (changed from reasonable response) were observed [31].

Synaptojanin-1 (*SYNJ1*) mutations cause autosomal recessive juvenile atypical parkinsonism (PARK20). *SYNJ1* encodes synaptojanin-1, a protein with two phosphatase domains (SAC and INPP5c), an uncommon feature in the human proteome; another example is LRRK2. The SAC domain is particularly relevant, with the recurrent *R258Q* mutation causing parkinsonism. The first report described an Italian family with autosomal recessive early-onset parkinsonism, detected by homozygosity mapping, in which the levodopa response was variable [32]. The same homozygous Arg258Gln mutation was later identified on different haplotypes, suggesting a mutational hotspot [25]. *SYNJ1* mutations can also cause seizures and developmental delay, particularly with homozygous p.R136X mutations that produce a near-complete loss of function [33,34]. Partial *SYNJ1* function, by contrast, is typically associated with parkinsonism alone. Some pathological reports have also shown tau-immunoreactive neurofibrillary degeneration [35].

*DNAJC6* and *SYNJ1* act sequentially in the same pathway of clathrin disassembly, a fundamental process for endosomal formation, vesicle trafficking, and membrane recycling. *SYNJ1* dephosphorylates phosphatidylinositol 4,5-bisphosphate (PI(4,5)P2), removing adaptor proteins from the inner cell membrane. *DNAJC6*, together with auxilin and HSP70, then promotes clathrin uncoating. This recycling process allows vesicles to be reused in the synaptic cleft or redirected to other pathways [36]. Remarkably, every cell in the body recycles its entire membrane approximately every fifteen minutes.

Another critical protein regulating vesicle trafficking and endosomal pathways is *RAB39B*, mutations in which cause intellectual disability and early-onset Parkinson's disease with α-synuclein pathology, and in some cases, tau pathology [37]. A hemizygous missense mutation in *RAB39B* was identified in affected members of the Wisconsin kindred with Waisman syndrome, where patients were levodopa-unresponsive and carried a Thr168Lys mutation [38]. Within the RAB protein family, RAB32 has also been implicated in late-onset Parkinson’s disease, showing reduced penetrance; notably, *RAB32* is phosphorylated by LRRK2 [39].

Collectively, mutations in *DNAJC6*, *SYNJ1*, and *RAB39B* highlight the particular vulnerability of synaptic vesicle trafficking pathways, showing how disruptions first manifest as developmental delay or juvenile parkinsonism but may later converge on mechanisms central to late-onset neurodegeneration.

PSMF-1

A recent discovery is the proteasome inhibitor subunit 1 (PSMF1) variants leading to early-onset Parkinson's disease with disruption of the mitochondria integrity (morphology, potential, and fragmentation). The PSMF1 protein is a proteasome regulator that involves the quality control of proteins and is known to interact with the F-box only protein 7 (FBXO7), the gene causing PARK15. Therefore, PSMF1 and PARK15 share some characteristics. The phenotype widely varies from arthrogryposis during infancy and global hypokinesia in mid-childhood to parkinsonism during adolescence and early adulthood. The patients who developed parkinsonism had a good response to levodopa, but most had parkinsonism with atypical signs [40].

Shared Genetic Risk Factors

Shared genetic risk factors between NDDs and neurodegenerative diseases highlight the complex genetic interplay influencing brain function across the lifespan. Chromosomal abnormalities, such as trisomy 21 in Down syndrome, significantly increase the risk of Alzheimer's disease due to overexpression of the *APP* gene, leading to early β-amyloid accumulation and accelerated neurodegeneration [41]. Single-gene mutations also contribute to both early neurodevelopmental impairment and late-onset decline. Mutations in *TSC1* and *TSC2*, which cause tuberous sclerosis complex, disrupt mTOR signaling, leading to neuronal dysregulation, epilepsy, and an increased risk of tauopathy [42]. Similarly, *MECP2* mutations in Rett syndrome affect transcriptional regulation and synaptic plasticity, contributing to early developmental regression and progressive motor deterioration resembling parkinsonian features [43]. Beyond monogenic disorders, polygenic risk factors identified through genome-wide association studies (GWAS) reveal overlapping susceptibility loci between NDDs and neurodegeneration, implicating pathways involved in synaptic maintenance, neuroinflammation, and protein homeostasis.

Epigenetic Mechanisms and Neurodegeneration

Epigenetic modifications play a crucial role in regulating gene expression throughout neurodevelopment and aging, with significant implications for late-onset neurodegeneration in individuals with NDDs [44]. DNA methylation, primarily occurring at cytosine-phosphate-guanine (CpG) sites, is essential for neuronal differentiation and synaptic plasticity, yet aberrant methylation patterns contribute to neurodevelopmental abnormalities and subsequent neurodegeneration [45]. For example, emerging evidence indicates that hypermethylation of the brain-derived neurotrophic factor (BDNF) promoter is observed not only in Rett syndrome and Alzheimer's disease but also in Parkinson's disease, major depressive disorder, and schizophrenia, where it has been linked to impaired synaptic function, reduced neurotrophic support, and heightened neuronal vulnerability [46-50]. However, these associations remain correlational, and the precise causal mechanisms may differ across conditions, warranting cautious interpretation. Similarly, histone modifications, such as acetylation and methylation, regulate chromatin accessibility and gene transcription, with dysregulation linked to neurodegenerative diseases like frontotemporal dementia [51]. Epigenetic mechanisms also mediate environmental influences on neurodevelopment and neurodegeneration. Factors such as prenatal stress, toxin exposure, and diet can induce lasting epigenetic changes, increasing vulnerability to late-onset neurodegeneration. These findings underscore the importance of investigating epigenetic interventions as potential therapeutic strategies for individuals with NDDs at risk of progressive neurological decline.

Mitochondrial Dysfunction and Oxidative Stress

Mitochondrial dysfunction is increasingly recognized as a shared pathological mechanism between NDDs and neurodegenerative disorders [52]. Mitochondrial DNA (mtDNA) mutations disrupt oxidative phosphorylation, impairing ATP production, increasing reactive oxygen species (ROS), and leading to neuronal energy deficits. In NDDs such as Leigh syndrome and mitochondrial encephalopathies, early-onset neurodevelopmental impairments often precede progressive neurodegeneration. Similarly, mutations in genes regulating mitochondrial function, such as *PINK1* and Parkin, have been implicated in developmental brain abnormalities and late-onset neurodegenerative diseases like Parkinson's disease [53]. Beyond genetic mutations, oxidative stress resulting from mitochondrial dysfunction exacerbates neuronal damage, contributing to synaptic degeneration and white matter abnormalities in conditions such as Rett syndrome and Fragile X-associated tremor/ataxia syndrome (FXTAS). However, it remains unresolved whether oxidative stress acts as a primary driver initiating neurodegenerative cascades or emerges as a secondary consequence of pre-existing cellular dysfunction [54]. Addressing this causality gap is crucial, as it will shape whether therapeutic efforts should aim to prevent early ROS generation or to enhance cellular resilience after damage has occurred. The metabolic dysregulation observed in NDDs and neurodegeneration suggests that targeting mitochondrial resilience and redox balance may offer neuroprotective strategies for individuals at risk of progressive neuronal decline.

Early developmental phenotype

The early developmental phenotype in NDDs is characterized by distinct patterns of atypical development across motor, cognitive, and behavioral domains. These phenotypes typically emerge during infancy or early childhood and are pivotal for early diagnosis and intervention. Delayed motor milestones are typical in Rett syndrome, reflecting underlying abnormalities in neural circuitry and muscle tone [55]. Similarly, cognitive delays, impaired communication, and atypical social behaviors are hallmark features in autism spectrum disorder (ASD) and intellectual disability, highlighting disruptions in brain regions responsible for social cognition, language development, and executive functions [56].

A complex interplay of genetic and environmental factors shapes early developmental phenotypes. For instance, mutations in genes such as *MECP2*, *CDKL5*, and *FMR1* result in distinct neurodevelopmental trajectories due to their critical roles in synaptic plasticity and neuronal signaling [57]. Environmental influences, including prenatal exposure to toxins, infections, or nutritional deficiencies, can further exacerbate developmental delays and amplify vulnerabilities. These phenotypes serve as diagnostic markers and offer invaluable insights into the disrupted biological pathways in NDDs. Characterizing the early developmental phenotype is essential for identifying at-risk individuals, optimizing early interventions, and predicting long-term outcomes. Furthermore, understanding these early features provides a foundation for investigating how neurodevelopmental disruptions evolve into neurodegenerative processes.

Cognitive and Behavioral Features

Several NDDs present with early-onset cognitive and behavioral impairments, subsequently progressing to late-onset neurodegeneration. One such disorder is Tay-Sachs disease, a lysosomal storage disorder caused by mutations in the *HEXA* gene, leading to the accumulation of GM2 gangliosides within neurons. Infants with Tay-Sachs disease typically appear normal until approximately six months of age, after which they exhibit developmental regression, loss of motor skills, and increased startle response [58]. As the disease advances, affected children experience seizures, vision and hearing loss, cognitive decline, and paralysis, culminating in death, usually before the age of four.

Another example is Batten disease, also known as neuronal ceroid lipofuscinosis (NCL), a group of autosomal recessive disorders characterized by the accumulation of lipofuscins in the body's tissues. Children with Batten disease often present between the ages of 5 and 10 with vision problems or seizures, followed by progressive cognitive decline, behavioral changes, and loss of motor functions. As the disease progresses, individuals may become blind, bedridden, and lose all cognitive functions, with life expectancy varying depending on the specific form of NCL [59].

FXTAS is another disorder that illustrates the progression from early cognitive and behavioral features to late-onset neurodegeneration. FXTAS affects carriers of the *FMR1* gene premutation and typically manifests after age 50. Early signs include intention tremor and gait ataxia, which may be accompanied by parkinsonism, cognitive decline, and autonomic dysfunction [60]. Over time, these symptoms progress, leading to significant impairment in daily functioning.

Motor and Neurological Features

Parkinsonism is clinically characterized by bradykinesia and tremor or rigidity. Early-onset parkinsonism is defined by the onset of parkinsonism in an individual less than 40 years old, and it can be divided into juvenile parkinsonism (< 21 years) and young-onset parkinsonism (21-40 years) [61]. Parkinsonism in children has a heterogeneous etiology, frequent co-occurrence of a hyperkinetic disorder, additional neurological or systemic findings, and varied associated neuroimaging findings. In this way, parkinsonism in children is divided into developmental parkinsonism, infantile and early childhood degenerative parkinsonism, parkinsonism in the setting of NDDs, parkinsonism in the setting of multisystemic brain diseases, juvenile parkinsonism and dystonia parkinsonism, and acquired parkinsonism [62].

Cases of developmental parkinsonism are not true neurodegenerative, and this can be supported by the fact that cases of patients who did not receive treatment do not develop neurodegenerative features. An example is GTP cyclohydrolase (GTPCH-I), also known as Segawa syndrome, which shows a dramatic response to levodopa. Another example is PTPS (6-pyruvoyl-tetrahydropterin synthase) deficiency, which also exhibits the hallmarks of BH4 (tetrahydrobiopterin) deficiency, including hypotonia, impaired motor and cognitive development, and parkinsonism. The level of deficiency of tyrosine hydroxylase (TH) explains the phenotype varying from mild (dystonia) to severe (parkinsonism with motor delay) to very severe (progressive infantile encephalopathy). A fourth enzyme important in the metabolism of monoamines is the AADC (aromatic L-amino acid decarboxylase) [63,64].

The WARS2 (tryptophanyl tRNA synthetase 2) deficiency disorder affects the mitochondria. The WARS2 has a cytoplasmic (WAR) and a mitochondrial (WARS2) form, which is crucial for mitochondrial translation, especially in tissues with high energy demands, such as the central nervous system. WARS2 deficiency is phenotypically differentiated between epilepsy and movement disorder. The movement disorder phenotype typically starts in childhood or early adulthood and is associated with a hypomorphic WARS2 variant c.37T>G (p.Trp13Gly) [65].

Late degenerative phenotype

Neurodevelopmental disorders can be associated with later neurodegenerative disorders. The phenotypic changes over the course of the disease appear to influence the neurodegenerative process. However, development can influence the movement disorder phenotype, and the development of encephalopathies and movement disorders may be both part of a phenotypical continuum.

In the typical form of glutaric aciduria type 1, encephalopathy occurs in the first years of life, often accompanied by striatal necrosis. In many cases, the lesions remain relatively stable; however, despite this stability, some patients experience a change in their phenotype. Usually, young patients with the glutaric aciduria type 1 phenotype are characterized by hyperkinetic (choreoathetosis) movements with mobile dystonia. As they become older during adulthood, they can develop fixed dystonia and akinetic-rigid parkinsonism, even with stable central nervous system lesions [66]. Therefore, development can influence the movement disorder phenotype even in the absence of macroscopic neurodegeneration.

Similarly, in myoclonus-dystonia, which is considered a non-neurodegenerative condition, some patients exhibit changes in phenotype over time, with dystonic features improving while myoclonus emerges or intensifies during aging [67]. Another example is Rett syndrome, where motor and cognitive phenotypes evolve from a hyperkinetic to a more hypokinetic state despite largely stable structural brain findings [68]. In Tourette syndrome, tic severity often decreases after adolescence, even though no progressive neurodegeneration is seen [69]. These examples support that significant phenotype shifts can occur without evidence of macroscopic neurodegeneration.

Movement disorders and developmental encephalopathies may both be part of a phenotypical continuum. For example, in dystonia, abnormal sensory input occurs during critical developmental windows preceding the onset of dystonia [70]. Additionally, monogenic dystonia is associated with early defects in neuritogenesis and synaptic plasticity [70]. Additionally, enhanced neuroplasticity during development can alter the "set point" of synaptic activity [70]. Furthermore, a molecular intersection may exist between dystonia and developmental encephalopathies [70]. Therefore, changes in the phenotype of movement disorders during infancy and childhood may not necessarily indicate neurodegeneration.

MECP2-Related Developmental Encephalopathy (Rett Syndrome)

*MECP2* is a gene that encodes the methyl-CpG-binding protein 2 (MeCP2), which modulates gene expression (including control of neuronal transcriptomic profile). Its role in neurons regulates various aspects of neural development, especially neurogenesis and synaptic connections. The developmental phenotype is initially normal, with psychomotor regression before the age of two, with intellectual disability, prominent apraxia, hypotonia, stereotypic movements (mouth and hand), epilepsy, and acquired microcephaly. On the other hand, the late phenotype is characterized by akinetic-rigid parkinsonism (freezing postural instability) and dystonia, which gradually increase in frequency with aging and are frequently seen during adulthood. In this context, the severity of the dystonia in patients with *MECP2 *disorder was already associated, independently of the body part, with the aging process [71], homovanillic acid levels in cerebrospinal fluid, iron accumulation in the dopaminergic network and gray matter correlation, and reduction in the density of dopamine D2 receptors (D2Rs) in the striatum [72-74].

SCN1A-Related Developmental Encephalopathy (Dravet Syndrome)

*SCN1A* is a gene that provides instructions for making the alpha subunit of the voltage-gated sodium channel NaV1.1, in which its alterations may impair sodium currents and action potential firing, especially in the hippocampus and cerebellum, leading to reduced GABAergic neurotransmission and hyperexcitability. The developmental phenotype includes epileptic encephalopathy characterized by severe early-onset epilepsy, developmental delay, and intellectual disability. The late phenotype partially responds to dopa in akinetic-rigid parkinsonism cases [75], with axial predominance and dystonic features (one-third exhibit a crouch gait) and gradually worsening gait and parkinsonism with aging [76].

FOXG1-Related Developmental Encephalopathy

The Forkhead box G1 (*FOXG1*) gene is responsible for producing a transcription factor, which has a pleiotropic role in brain development (cell proliferation and progenitor pool expansion, regional patterning of the forebrain, cell migration during corticogenesis, and circuit assembly). The developmental phenotype has encephalopathy with severe early-onset developmental delay, intellectual disability, epilepsy, psychiatric disorders, stereotypies, microcephaly, postnatal growth deficiency, and hyperkinetic movements. The late phenotype has dystonia and parkinsonism in some patients, and late-onset hyperkinetic movements can also be observed [77].

22q11.2 Deletion Syndrome (DiGeorge Syndrome)

22q11.2 deletion syndrome is a genetic condition that results from the deletion of a small portion of chromosome 22. Multiple system disorders with developmental issues, including congenital malformations (heart, palate, kidney, and gastrointestinal), immune disorders with recurrent infections, endocrine disorders (hypocalcemia), epilepsy, developmental delay, intellectual/learning disability, and psychiatric disorders. Also, the late phenotype is characterized by early-onset (typically 30-45) Parkinson's disease with clinical and neuropathological characteristics similar to those of idiopathic Parkinson's disease [78]. Some of the proposed mechanisms are hyperdopaminergic mechanisms with dopamine autotoxicity related to catechol-o-methyltransferase (COMT) haploinsufficiency, mitochondrial dysfunction with several genes involved in mitochondrial function located within the deletion area, and overexpression of alpha-synuclein in the striatum based on data from animal models [79].

PPP2R5D-Related Developmental Encephalopathy

The late phenotype is characterized by early-onset (20-50 years of age) dopa-responsive parkinsonism and adult-onset parkinsonism, which can rarely be isolated. The developmental phenotype is characterized by psychomotor delay, intellectual disability, ASD, macrocephaly, epilepsy, and visual impairment. In the cases of PTPA (PPP2R4)-related intellectual disability, the late phenotype is characterized by early-onset dopa-responsive (adolescence) Parkinson's disease [28].

RAB39B-Related Developmental Encephalopathy

The developmental phenotype of RAB39B disorder is characterized by psychomotor delay, intellectual disability, neuropsychiatric disorder, and epilepsy. The late phenotype has early-onset (20-40 years old) dopa-responsive parkinsonism or dystonia parkinsonism [80].

NR4A2-Related Developmental Encephalopathy

NR4A2, or nuclear receptor 4A2, is a transcription factor that plays a role in the differentiation and maintenance of neurons (especially midbrain dopaminergic). The developmental phenotype has psychomotor delay, intellectual disability with prominent speech/language deficits, neuropsychiatric disorders, and epilepsy. The late phenotype has early-onset (20-40 years old) dopa-responsive parkinsonism or dystonia parkinsonism (isolated dystonia) [81].

Brain structures involved in neurodevelopmental and neurodegenerative disorders

Cognitive decline in Alzheimer's disease appears to follow a pattern opposite to the developmental acquisition of cognitive abilities in childhood and adolescence. Similarly, neurodegenerative disorders affect brain structures in a characteristic sequence that mirrors, in reverse, the ontogenetic development of the neocortex, particularly in the temporal, parietal, and frontal lobes. During early development, infants exhibit high levels of phosphorylated tau, a process essential for axonal destabilization and synaptic plasticity, both of which are crucial for brain maturation and neuroplasticity [82]. Neurofibrillary tangles primarily accumulate in brain regions that take the longest to mature, particularly the temporal and frontal lobes [83]. These areas, integral to complex cognitive functions, including language, memory, perception, self-awareness, and consciousness, retain a degree of neuroplasticity throughout adulthood. Notably, in both neurodevelopmental and neurodegenerative disorders, including Alzheimer's disease and synucleinopathies, the same brain structures exhibit early cortical developmental abnormalities and later neurodegenerative pathology [53].

The selective vulnerability of cortical and subcortical structures plays a critical role in NDDs and late-onset neurodegeneration. The temporal, parietal, and frontal lobes, essential for cognitive functions such as memory, executive processing, and language, are frequently affected in conditions ranging from ASD to Alzheimer's disease. In neurodevelopment, impaired cortical maturation due to genetic mutations or environmental insults can lead to structural and functional deficits predisposing individuals to later neurodegeneration. Similarly, the basal ganglia, a key motor control and learning regulator, is commonly affected in movement disorders that span NDDs and neurodegenerative conditions. For example, mutations in *GNAO1* and *ATP1A3* disrupt basal ganglia function in developmental dystonia, while dysfunction of the same circuitry underlies parkinsonian features in disorders such as FXTAS and Huntington's disease. This shared vulnerability suggests that early structural and functional abnormalities may create a predisposition for progressive neural decline later in life.

Synaptic dysfunction represents a crucial link between NDDs and late-onset neurodegeneration, as both processes involve synaptic plasticity and connectivity alterations. Synaptic pruning, a highly regulated process during early brain development, is necessary for refining neural circuits; however, excessive or deficient pruning has been implicated in disorders such as schizophrenia and ASD. In contrast, late-life synaptic loss is a hallmark of neurodegenerative diseases like Alzheimer’s and Parkinson’s disease, where dysregulated synaptic maintenance leads to cognitive decline and motor dysfunction. For example, altered glutamatergic and GABAergic signaling in NDDs can persist into adulthood, contributing to excitotoxic damage and progressive neuronal loss. The bidirectional relationship between synaptic plasticity and neurodegeneration highlights the importance of understanding molecular regulators such as BDNF and microglial-mediated synaptic remodeling, which could serve as therapeutic targets for mitigating cognitive decline.

White matter integrity is essential for neural communication and is frequently compromised in NDDs and neurodegenerative diseases. Myelination deficits in NDDs, such as those seen in Pelizaeus-Merzbacher disease and hypomyelinating leukodystrophies, impair signal transmission and neural network efficiency, often manifesting as cognitive and motor impairments. Similarly, late-onset neurodegenerative disorders, including multiple system atrophy and leukodystrophies, exhibit progressive demyelination, leading to widespread neural dysfunction. Disruptions in axonal connectivity and white matter tract integrity have been observed in both ASD and frontotemporal dementia, suggesting a continuum of vulnerability that spans neurodevelopment and aging. Advanced neuroimaging techniques, such as diffusion tensor imaging (DTI), have revealed overlapping patterns of white matter abnormalities in individuals with early-onset neurodevelopmental conditions and those with neurodegenerative diseases, underscoring the importance of white matter pathology in long-term neural health.

Biomarkers and predictive models of late-onset neurodegeneration

Genetic and epigenetic biomarkers offer critical insights into the prediction and progression of late-onset neurodegeneration in individuals with NDDs. Among these, genetic variants with well-established clinical utility, particularly Alzheimer's disease-linked mutations in *APP*, *PSEN1*, and *PSEN2*, as well as *LRRK2* and *GBA* mutations in Parkinson's disease, show the strongest translational potential because they are already used in genetic risk stratification and counseling [84,85].

In contrast, epigenetic markers such as DNA methylation and histone modifications are emerging tools that reflect biological aging and may serve as early indicators rather than definitive predictors [86]. Age-related methylation shifts, assessed through "epigenetic clocks," and alterations in genes regulating neuroinflammation, synaptic plasticity, and oxidative stress have been implicated in both NDDs and neurodegeneration [87], highlighting the interplay between early genetic predispositions and late-life neuropathology.

Proteomic and metabolomic markers also provide insights, but their translational readiness varies. Proteinopathies involving well-characterized misfolded proteins (e.g., tau, β-amyloid, and α-synuclein) have direct diagnostic and therapeutic relevance in late-onset neurodegenerative diseases [88,89].

In individuals with NDDs, early disruptions in protein homeostasis (e.g., mTOR dysregulation in tuberous sclerosis complex) are promising but less validated biomarkers [90].

Similarly, persistent neuroinflammatory signatures such as elevated IL-6 and TNF-α levels have been reported in Rett syndrome and Fragile X syndrome [91], but their causal link to later neurodegeneration remains to be confirmed.

Metabolomic markers of oxidative stress and mitochondrial dysfunction are highly promising for early detection but still exploratory [92]. Neuroimaging and electrophysiological markers currently hold the most immediate clinical applicability for longitudinal monitoring. Functional and structural magnetic resonance imaging (MRI) studies have revealed replicable patterns of cortical thinning and white matter disruption in ASD and Alzheimer's disease [93,94]. Additionally, DTI has demonstrated that early white matter abnormalities can predict later decline [95].

Moreover, electroencephalography (EEG) markers, such as altered spectral power and increased epileptiform activity, observed in Dravet syndrome and Rett syndrome, as well as dementia with Lewy bodies and Alzheimer's disease [96,97], are feasible and noninvasive tools with strong translational potential for early surveillance.

Future studies

Future studies should continue to evaluate late-onset phenotypes of developmental encephalopathies, as they hold significant clinical relevance. The late worsening of these conditions contributes to increased disability and reduced autonomy, often going undiagnosed, with detrimental consequences for patients. In particular, priority should be given to phenotypes involving progressive cognitive decline (e.g., dementia-like presentations), emerging motor syndromes (e.g., parkinsonism, ataxia), and chronic neuropsychiatric disturbances (e.g., depression, psychosis, sleep dysregulation) in individuals with Rett syndrome, Fragile X syndrome, and tuberous sclerosis complex, as these domains are strongly associated with neurodegenerative trajectories [98-100].

Additionally, individuals with developmental encephalopathies should be systematically monitored for neurodegenerative progression during follow-up visits to facilitate early intervention and tailored management strategies. Longitudinal monitoring protocols should incorporate standardized cognitive batteries, quantitative motor assessments, and neuropsychiatric screening tools to detect early decline in these domains [101,102].

Longitudinal cohort studies are essential for identifying the inflection point of neurodegeneration in specific genetic conditions. However, the rarity of these disorders and the economic burden of genetic investigations present challenges to large-scale studies. Advancements in next-generation sequencing and multi-omics approaches could enhance the feasibility of these studies, allowing for a more comprehensive understanding of genotype-phenotype correlations and disease trajectories.

Further research should focus on identifying early biomarkers of neurodegeneration in these populations. Among the currently proposed indicators, genetic variants and neuroimaging abnormalities have the strongest evidence base, with several longitudinal studies linking risk alleles (e.g., APOE ε4) and structural or functional MRI changes to later onset of neurodegenerative disease [103,104]. Epigenetic modifications show growing but still emerging evidence, as alterations in DNA methylation and histone acetylation have been associated with neurodegenerative risk but remain largely correlative [105]. Proteomic signatures are highly promising yet remain largely speculative, as most findings come from small exploratory cohorts without consistent replication [106]. The integration of machine learning and artificial intelligence in biomarker discovery may help combine these modalities into predictive models, potentially improving early diagnosis and risk stratification.

Therapeutic approaches should also be explored to mitigate the progression of late-onset neurodegeneration in individuals with NDD. For example, recent preclinical studies using adeno-associated virus-mediated delivery of *MECP2* in Rett syndrome models have demonstrated partial rescue of neurological deficits [107]. Early-phase clinical trials of antisense oligonucleotides targeting UBE3A reactivation are currently underway for Angelman syndrome [108].

Gene therapy, targeted molecular treatments, and neuroprotective interventions hold promise, but their application requires a deeper understanding of disease mechanisms. Additionally, small-molecule modulators such as mTOR inhibitors (e.g., Everolimus) have demonstrated neurocognitive benefits in individuals with tuberous sclerosis complex [109], illustrating the potential of disease-specific neuroprotective strategies. Collaborative efforts between geneticists, neurologists, and data scientists will be crucial in translating these findings into clinical practice, ultimately improving patient outcomes.

## Conclusions

Late-onset neurodegeneration in individuals with NDDs represents a continuum linking early-life vulnerabilities to progressive neurological decline. Genetic, epigenetic, and environmental factors interact to shape the early developmental phenotype, which may predispose the brain to later degenerative processes. Mechanisms such as synaptic dysfunction, mitochondrial impairment, oxidative stress, and neuroinflammation serve as common pathways bridging neurodevelopment and neurodegeneration. Identifying predictive biomarkers through genomic, proteomic, and neuroimaging approaches offers the potential for early detection and risk stratification. Longitudinal studies and experimental models are essential to delineate the trajectory from developmental abnormalities to neurodegenerative outcomes. Understanding these mechanisms could enable the development of timely interventions, including targeted therapies and neuroprotective strategies, during critical developmental windows. By integrating developmental and degenerative research, we can improve prognosis, inform personalized clinical management, and ultimately reduce the lifelong burden of these complex disorders.
